# Black Bean Pasta Meals with Varying Protein Concentrations Reduce Postprandial Glycemia and Insulinemia Similarly Compared to White Bread Control in Adults

**DOI:** 10.3390/foods11111652

**Published:** 2022-06-03

**Authors:** Donna M. Winham, Sharon V. Thompson, Michelle M. Heer, Elizabeth D. Davitt, Sharon D. Hooper, Karen A. Cichy, Simon T. Knoblauch

**Affiliations:** 1 Department of Food Science & Human Nutrition, Iowa State University, Ames, IA 50011, USA; mmheer@gmail.com (M.M.H.); eddavitt23@gmail.com (E.D.D.); simon.t.knoblauch@gmail.com (S.T.K.); 2 Division of Nutritional Sciences, University of Illinois at Urbana-Champaign, Champaign, IL 61801, USA; sharonvt83@gmail.com; 3 Department of Plant, Soil and Microbial Sciences, Michigan State University, East Lasing, MI 48824, USA; hoopers1@anr.msu.edu; 4 Sugarbeet and Bean Research, USDA-ARS, East Lansing, MI 48824, USA; karen.cichy@ars.usda.gov

**Keywords:** pulses, legumes, plant-based foods, pasta, glycemic response, gluten free, satiety, flatulence

## Abstract

Postprandial glycemic and insulinemic effects of three black bean pastas were evaluated among eighteen normoglycemic adults (8 men, 10 women) in a randomized crossover trial. Black beans were milled into flour using a commercial Knife or compression/decompression mill (C/D mill). The C/D-mill-derived pastas had medium protein (Combo-MP) and low protein (Cyclone-LP) concentrations. Three black bean flour pastas (Knife, Combo-MP, and Cyclone-LP) were compared to two controls: white bread and whole black beans. Treatments contained 50 g of available carbohydrate. Plasma glucose, serum insulin, and appetite measures were collected at fasting and 30, 60, 90, 150, and 180 min postprandial. Gastrointestinal symptoms were evaluated 10–12 h postprandial. ANOVA (one-way, repeated measures) was used to evaluate satiety, gastrointestinal symptoms, sensory variables, glucose and insulin differences from baseline, and incremental area under the curve (iAUC) by time and/or treatment. Three-hour glucose and insulin iAUCs were lower with whole black beans than white bread. Black bean pasta meals increased satiety, reduced appetite, and produced numerically lower glucose and insulin responses than white bread. However, no differences were observed between pasta types, indicating a similar metabolic response regardless of milling technique. Our results provide evidence for dietary guidance to reduce postprandial glucose and related health risks through pulse food products.

## 1. Introduction

Beginning in the late 1920s, pasta started its ascent as one of the most popular foods in the United States (US) [1]. A majority of US consumers (86%) report weekly pasta consumption [2]. The US generates around 2.0 million kilograms of pasta annually and is the second largest pasta producer in the world [2]. Traditionally, pasta is made from durum wheat semolina, which has been valued for its high gluten and quality characteristics. Pasta consumers ate more fiber, food folate, and certain minerals than non-consumers in one report [3]. However, despite their widespread consumption, high-gluten refined wheat pastas have been critiqued for their low fiber and protein content as well as their possible reactivity among gluten-intolerant or gluten-sensitive individuals. Celiac disease prevalence has increased by nearly 8% each year over the past several decades [4,5]. There has also been a heightened interest in gluten-free (GF) foods beyond their use for medical reasons. GF foods are often perceived to be healthier than their conventional counterparts. However, many GF pastas are made from refined starches of comparatively low nutrient density and high glycemic index [6].

Many factors, such as an increased consumer interest in GF foods, high protein foods, plant-based diets, and environmental sustainability have led to greater interest in pulse-based or pulse blend pasta formulations in recent years [6,7,8]. Pulses are a subcategory of legumes grown for use of their seeds as dry grains in many cultures globally. Within the pulse category are dry beans, dry peas, lentils, chickpeas, and black-eyed peas. Pulses are nutrient dense, providing plant sources of protein, carbohydrates, dietary fiber, minerals such as iron, zinc, calcium, and magnesium, vitamins such as folate, riboflavin, and thiamin, and phytochemicals [9,10]. Foods high in fiber such as pulses are linked to improved satiety and reduced appetite in healthy adults [11]. Pulse consumption is also connected with the prevention and treatment of chronic conditions such as hyperlipidemia, metabolic syndrome, type 2 diabetes mellitus (T2DM), hypertension, and coronary heart disease [12,13,14,15]. Long-term pulse consumption is associated with a lower risk of certain cancers, improved glycemic control, and increased satiety which can play a role in weight management and increased longevity [15,16,17,18,19].

Despite the many known benefits of pulse consumption, US intakes are historically lacking. The Dietary Guidelines for Americans (DGA) recommends legume consumption (beans and peas) of 1.5 cup equivalents per week for a 2000-calorie diet [20]. In a recent evaluation of the 2-day diet records from the National Health and Nutrition Examination Survey (NHANES) for 2004–2014, consumption was consistently below recommended levels and remained so over the study period [21]. Incorporation of pulses into popular consumer products may offer a more appealing and accessible way to both increase pulse consumption and improve diet quality.

Dry bean pastas have greater protein, bioactive compounds (total polyphenols and flavonoids), antioxidant properties, as well as higher resistant starch and lower total starch content than wheat pastas [7,22,23]. Pastas prepared with pulse flours are more nutritionally desirable from a health standpoint than other GF pastas due to their consistently lower glycemic index and net glucose response [22,23,24,25,26]. Adding beans to foods such as pastas offers a convenient and palatable method to raise pulse consumption for many US households. Increased interest in consumption of GF foods also makes bean pastas a desirable food product.

Whole boiled beans produce a low postprandial glycemic response by themselves and as part of a meal among normoglycemic adults and those with type 2 diabetes (T2DM) [17,27,28,29]. A blunted glycemic response following bean consumption is due, in part, to the strong cotyledon cell wall which remains intact during cooking and reduced surface area for human digestive-enzyme-related degradation [30,31]. Beans induce a higher glycemic response when the cell wall is degraded, as occurs with bean flour, than if they are provided in a whole bean form with an intact cell wall [32]. When raw beans are milled into flour, their cellular components are more accessible to digestive enzymes and contain more free starch, which resulted in a higher blood glucose response than whole boiled beans in one comparison [33].

Demand for pulse flours as ingredients in processed food has increased in the US and internationally in recent years. In the US, a 20% growth in pulse flour pasta demand is expected by 2023, a percentage higher than what is projected for wheat [34]. Pulse milling is a new industry without clear particle size specifications and target protein concentrations [35]. Many milling innovations are being explored in pulses to achieve desired particle sizes, reduce off-flavors and starch damage, and modulate the protein and fiber levels [36]. It is possible that different milling techniques will influence the glycemic response of foods made from the resulting flours.

The objective of this study was to determine the effect of three 100% black bean pastas made from differently processed flours on postprandial metabolic response, appetite measures, and gastrointestinal symptoms in normoglycemic adults in comparison to white bread and boiled whole black bean controls. The flour milling techniques were a standard process (Knife mill) and a novel milling method of compression/decompression (C/D mill). We hypothesized that postprandial glucose, insulin, appetite, and gastrointestinal symptom measures would differ between the bean pastas and control treatments.

## 2. Materials and Methods

### 2.1. Study Recruitment and Selection Criteria

Potential participants were recruited through a campus email sent to students and employees. Interested persons completed an online screening form to determine pre-eligibility for a second in-person screening with a blood draw. The initial selection criteria included age 21–30 years, breakfast consumption on most days, not pregnant or lactating for women, being ambulatory, able to eat independently, willingness to eat beans and bean pasta, and agreeable to restrict vigorous exercise, caffeine, alcohol, or herbal teas for 24 h before testing. Exclusion criteria were weight instability (weight loss or gain > 4.5 kg in past 6 months), active intent to gain or lose weight, use of tobacco or nicotine delivery products, daily alcohol consumption, inability to get regular 6–8 h of sleep on evenings before testing, and lack of transportation to testing site. Those with restrictive dietary practices (low carbohydrate, low sodium, vegetarian, or vegan), food allergies, diabetes, or other medical conditions known to influence glycemic response study results were ineligible. If screening parameters were met, self-reported height and weight were used to determine if a respondent’s body mass index (BMI) was in the 20.0–29.9 kg/m^2^ range to advance to the second stage of in-person screening and a non-fasting blood draw.

Of the 217 screened via online assessment, 148 did not meet inclusion criteria. Sixty-nine people were invited to attend a 1 h information session on the study protocol, to learn compliance expectations, sign informed consent, provide a non-fasting blood sample, and have anthropometric measurements and blood pressure assessed. Fifteen people did not respond to the inquiry for further screening. Twenty-three had study screening or test date conflicts. During the information session, a team of researchers demonstrated how to complete the pre-test-day 24 h food logs, appetite measures survey, and gastrointestinal symptom questionnaire. Participants were instructed to limit food and liquid intake to plain water for 12 h prior to testing, to adhere to their typical sleep pattern, and refrain from exercise and alcohol consumption for 24 h prior to testing.

During the screening visit, participants completed the revised 18-item Three-Factor Eating Questionnaire to assess cognitive restraint of food intake. Individuals with a score >16 out of 24 on this subscale were disqualified [37]. For baseline behaviors, not screening, participants completed two validated food frequency screener questionnaires (FFQs) for (1) dietary fat and (2) for fruits, vegetables, and fiber [38]. Researcher-generated questions on consumption frequency of pasta, gluten-free foods, and pulses were asked at screening also (response categories: never, monthly, weekly, daily, multiple times daily).

Participants selected their pre-test evening meal choice. Selection was from three pre-approved frozen dinners and two cookie selections. The meal options were Marie Callender’s^®^ Honey Roasted Turkey, Salisbury Steak, or Roasted Turkey & Stuffing. The optional cookies were a Nabisco^®^ Teddy Grahams snack pack or a Pepperidge Farm^®^ Milano 2-pack. Participants were required to eat the same frozen dinner and cookie for all five pre-test evening meals. To ensure consistent carbohydrate intake before testing, participants were given plain bagels (Thomas^®^) and instructed to eat one daily for the three days preceding each testing day. This procedure was to ensure adequate glycogen stores to minimize any potential impact on postprandial glucose concentrations [8,27].

After participants provided written consent, a wall-mounted stadiometer was used to measure height to the nearest 0.1 cm (Seca Model 216, Chino, CA, USA). Body weight was measured to the nearest 0.1 kg on the screening day, as well as each subsequent test day using a digital scale (Detecto, Webb City, Missouri, USA). Waist circumference was evaluated with a non-elastic measuring tape to the nearest 0.1 cm (Seca 201, Chino, CA, USA). Blood pressure was measured twice using an Omron automatic digital blood pressure monitor (Omron Healthcare, Inc., Lake Forest, IL, USA) after participants sat quietly for 5 min. Elevated blood pressure was considered systolic blood pressure of ≥ 130 mm Hg, or diastolic blood pressure ≥ 80 mm Hg. No participants had high blood pressure. A registered nurse drew a non-fasting venous blood sample from the non-dominant arm into a 4 mL ethylene diamine tetra acetic acid (EDTA)-coated vacutainer. Samples were picked up by courier and analyzed for hemoglobin A1c (HbA1c) via an immunoturbidimetry assay, lipid panel, thyroid panel, and complete blood count by an automated hematology analyzer by a commercial laboratory (Quest Diagnostics, Wood Dale, IL). The first study test day was tentatively scheduled for participants after the screening blood draw. They were given their pre-test meal supplies, protocol instructions, and forms (pre-test-day 24 h food log, lunchtime appetite survey, and evening gastrointestinal symptom questionnaire).

Seven individuals were disqualified for abnormal long-term glucose concentration values (HbA1c < 4.6% or > 5.7%) (*n* = 4), iron deficiency anemia (*n* = 2), or other blood abnormalities (*n* = 1). Three of the seven were excluded as well by having Three Factor Eating Questionnaire cognitive restraint scores ≥ 16. Five individuals declined further participation for personal reasons (time conflicts; transportation). Nineteen participants started the study. One person had abnormal insulin responses (< 12.00 pmol/L postprandial) and was administratively withdrawn after two test days (data not shown). Data analysis from eighteen participants (8 men; 10 women) are presented. The study was approved by the Institutional Review Board at Iowa State University (#17-191-00). All participants provided written, informed consent. The clinical trial registry number for this study is NCT05182190.

### 2.2. Study Design

The randomized unblinded crossover trial included five treatments: (1) white bread (positive control); (2) whole black beans (negative control), and three 100% black bean pastas. The flour was derived from different milling technologies: (3) standard Knife mill; and two flour variants from a novel technology compression/decompression (C/D) system, (4) C/D mill with medium protein content, equivalent to that of whole beans (Combo—MP); and (5) C/D mill with lower protein (Cyclone—LP). A priori power analysis indicated that a sample size of 10 participants was sufficient for a medium effect size (d = 0.50) at 80% power with an α of 0.05 in this five-treatment, seven-timepoint crossover study.

### 2.3. Testing Protocol

#### 2.3.1. Test Day Procedures

On test day mornings, participants confirmed they were fasting and compliant with study procedures. Blood pressure and weight were recorded on each test day as described previously. After completion of these intake assessments, a registered nurse placed an indwelling catheter in the antecubital region of the participant’s non-dominant arm. Blood was drawn into an 8 mL serum separator vacutainer for glucose and insulin samples. Subsequently, participants received one of five randomly selected test meal options (Microsoft Excel, Redmond, WA, USA). Participants consumed the meal within 7–10 min under researcher supervision. Length of meal consumption time did not differ between treatments for each participant. Bottled water was provided ad libitum and the amount consumed over the 3 h test period was recorded. No other beverages or foods were allowed. Participants’ 24 h food logs were collected and reviewed by a research team member using the multi-pass dietary recall method [39].

Venous blood draws were collected at 30, 60, 90, 150, and 180 min post meal initiation. Samples sat for 30–60 min before being centrifuged in a Horizon Model mini E (Quest Diagnostics Inc., Secaucus, NJ, USA) at 3348 RPM for 10 min. Samples were promptly refrigerated pending Quest courier pick up later that day. Plasma glucose was determined by spectrophotometry. Serum insulin was assessed by immunoassay (Quest Diagnostics, Wood Dale, IL, USA).

#### 2.3.2. Subjective Appetite, Sensory, and Gastrointestinal Surveys

Subjective appetite questionnaires were completed each pre-test-day before and after the participant’s midday meal to familiarize them with the sensations they were feeling and to practice form completion. A researcher reviewed the pre-test-day forms during test day intake for protocol compliance. On test days, appetite perception measures were assessed before treatment consumption, and shortly after each blood draw at approximately 30, 60, 90, 150, and 180 min post meal initiation. Questions included hunger, fullness, satiation, desire to eat, and prospective consumption [40]. Responses were captured using a 100 mm visual analog scale (VAS) that was anchored with opposing statements (e.g., ‘not at all’ to ‘as much as I ever felt’). An overall appetite score was calculated as the mean of the following item scores: desire to eat, hunger, prospective consumption, and 100—fullness score [11].

Sensory scores were collected using a 9-point hedonic scale immediately after test meal consumption [41]. Participants ranked overall meal appearance, aroma, flavor, and texture, as well as the flavor and texture of the pasta sauce (dislike extremely/like extremely). A question on the size of the meal was anchored by scale responses of ‘extremely too little’ and ‘extremely too large.’ For the black bean pasta meals, participants were asked how likely it would be that they would purchase the pasta (extremely unlikely/extremely likely).

Participants completed two gastrointestinal (GI) symptom surveys (pre-test-day evening for practice, and the evening of the test day). Post-testing evening surveys were completed between 18:00 and 21:00. GI survey questions asked about changes in flatulence, bloating, stool frequency, stool consistency, and if symptoms interfered with normal daily activities [42,43]. Degree of change was noted as either increased or decreased using a scale from 0 to 5 representing ‘little change’ to ‘a lot of change’, or ‘a little more frequent’ to ‘much more frequent’.

#### 2.3.3. Test Meals

##### Pasta preparation

Black beans (Zenith) were heated at 110 °C for 70 min and milled into flour using a Knife mill (Comitrol, Model 1700, Urschel; sieve size 0.5 mm) or a C/D mill (Enagon, LLC, Saugatuck, MI, USA). The C/D mill by design generated bean flours with varying protein and fiber concentrations. The Knife mill flour and C/D mill combined flour (Combo-MP) both contained 26% protein, or ‘medium’ protein (MP). The second C/D mill flour tested had lower protein (LP) at 14% (Cyclone—LP). The flours were made into spaghetti (containing only 100% black bean flour) commercially by West Michigan Pasta and Provision, LLC (Kalamazoo, MI, USA). The fresh spaghetti was produced using a single screw extruder and then air dried at room temperature (25 °C) for 48 h.

##### Particle size distribution

**Figure 1:**
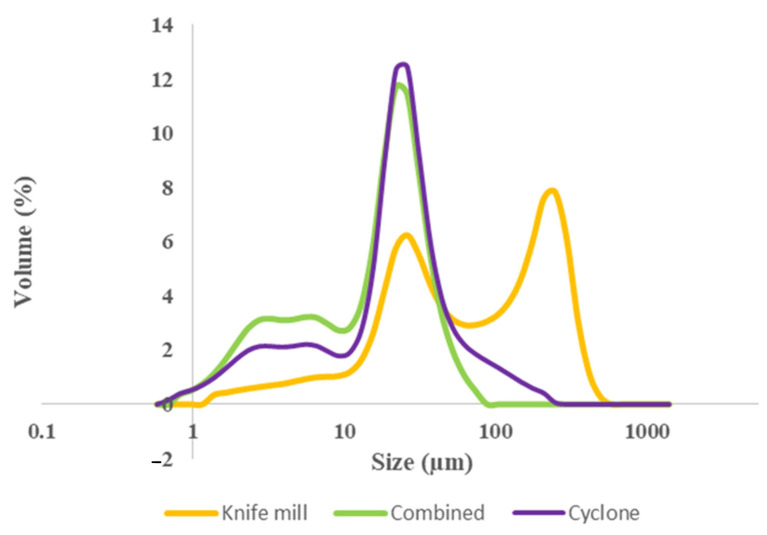
Particle size distribution of black bean flours milled using a Knife mill and a compression/decompression mill with resultant flours from Cyclone-LP (~14% protein) and Combo-MP (~24% protein) collection zones for pasta production. Results are means of duplicate analysis for each flour ingredient.

The three bean flours had different particle sizes (Table 1; Figure 1). Particle size distribution was determined as follows: bean flour samples (100 mg) were dispersed in 5 mL of propan-2-ol (99%) and stirred for 10 min. The dispersion was then analyzed, in triplicate, for particle size distribution by laser diffraction with a Microtrac S3500 (Microtrac Inc., North Largo, FL, USA). The results were expressed as d(0.1), d(0.5), and d(0.9), corresponding to the maximum diameters of 10%, 50%, and 90% of the particles, respectively, in % of total volume.

Figure 1 represents the curves of particle size distribution of whole bean flours milled via different technologies. Particle sizes ranged from 0.6 to 497.8 μm, while the mean diameter (average particle size) varied between 17.2 and 104.2 µm, with Knife milled flours having significantly larger average particle size than Combo-MP and Cyclone-LP milled flours. Particle size distribution is affected by mill type and can impact enzymatic starch digestion reactions. Smaller particle sizes can increase the bioavailability of macronutrients and thus glucose response [44].

The five test meals were white bread control (toasted, no sauce; Sara Lee Classic^®^), whole black beans, and three black bean pastas served with a standard amount of pasta sauce (Classico^®^ Traditional). Each test meal provided 50 g of available carbohydrates (total carbohydrates minus dietary fiber). Fifty grams of carbohydrates is the standard quantity used for glucose response testing [45]. The individual food components (white bread, pasta sauce, boiled whole black beans, three cooked pastas) were analyzed in duplicate for nutrient content by Eurofins (Des Moines, IA, USA). The test meals were similar in macronutrient content except for protein and fiber, which were greatest in the whole black bean meal (20.9 and 28.2 g) and lowest in the Cyclone-LP pasta (13.7 and 19.5 g) (Table 2).

Black bean pastas and whole black beans were prepared on test days in 2 L of boiling water with 15 g added salt. Optimum cooking time for each pasta was determined by the approved AACC method 66–50 [46]. Cooking times were 15 min for Knife mill, and 16 min for Combo-MP and Cyclone-LP. Whole black beans were soaked in water for 12 h the night before test days. After rinsing, the beans were boiled for 30 min. Commercial purified drinking water was used for all soaking and cooking procedures. Whole black beans and black bean pastas were weighed after cooking and draining. White bread and the pasta sauce were weighed before toasting and microwave heating, respectively. Treatment meal components were weighed to the nearest gram using a standard food scale (Mettler PC 4000, Toledo, Columbus, OH, USA). Test meals were served promptly after preparation. The white bread meal provided ~4.2 single slice servings (111.5 g) (Sara Lee, Horsham, PA, USA). The amount of pasta sauce served was ~7/8 of a cup (213 g) (International Gourmet Specialties, Pittsburgh, PA, USA). The cooked spaghetti ranged from 1.4–1.7 one cup servings [47]. The five test meals were consumed at least 4–7 days apart.

### 2.4. Statistical Analyses

FFQ responses, age, and gender were entered into the NutritionQuest online screener [48] to generate intake estimates of dietary fat percentage, fruit and vegetable servings, and grams of dietary fiber for each participant. Macro and micronutrient intakes from the five pre-test 24 h food logs were obtained using Food Processor (version 11.3, ESHA Research, Salem, OR, USA).

Data were analyzed for distribution normality by Shapiro–Wilk test and histogram evaluation. Variables that were not normally distributed were transformed using natural logarithm conversion. Mean imputation was employed for 23 missing serum insulin concentrations (approximately 4% of cases) due to hemolysis. GI responses were evaluated with Chi-square. Glucose and insulin iAUC values were calculated at 60, 120, and 180 min using the trapezoidal rule [49]. Glucose and insulin iAUC and sensory measures were analyzed with one-way ANOVA. General linear model testing for repeated measures was conducted to assess glucose, insulin, and subjective appetite score mean differences between treatments over time among normally distributed variables (raw or transformed). If unequal variances of differences were observed (Mauchly’s test *p* < 0.05), the Greenhouse–Geisser or Huynh–Feldt corrected tests of within-subject effects’ *p* value was used. Tukey HSD or LSD post hoc testing was then applied to pinpoint differences between specific treatments. SPSS version 27 (IBM, Armonk, NY, USA) was used for all statistical analyses.

## 3. Results

### 3.1. Participant Characteristics

Eighteen participants (8 men, 10 women; mean age 23.5 ± 0.6 years) completed the study. Participants were generally healthy with a mean BMI of 25.3 ± 0.7 kg/m^2^. All self-identified as White, and one person stated Hispanic ethnicity. Mean biomarker values for HbA1c (5.1 ± 0.5%), total cholesterol (4.3 ± 0.2 mmol/L), low-density lipoprotein cholesterol (LDL-C, 2.5 ± 0.1 mmol/L), high-density lipoprotein cholesterol (HDL-C, 1.3 ± 0.1 mmol/L), triglycerides (1.2 ± 0.2 mmol/L), hemoglobin (8.8 ± 0.3 mmol/L), and TSH (1.80 ± 0.1 ng/dL; data not shown) were within normal values. Due to participant scheduling conflicts, three participants completed four test treatments instead of five. Two participants did not consume whole black beans and one participant did not consume Combo-MP pasta.

### 3.2. Study Power

Observed power was between 0.98 and 1.00 for within-subject time by treatment difference in glucose concentrations, indicating at 98% minimum probability of making correct assumptions from these findings. More than half (56%) of the within-subject variation was associated with study time point, while treatment type comprised 17% of the variation with Greenhouse–Geisser correction. In contrast, between-subject (treatment type) power was 0.23. Multivariate testing with Wilks’ Lambda and α = 0.05 indicated significant effects between time points with a value of 0.165 and an observed power of 1.0. The partial eta squared was 0.84, signifying 84% of variability is based on the time points.

### 3.3. Food Frequency Questionnaires (FFQs) and 24 h Food Logs

The FFQ results for dietary fat and fruit, vegetable, and fiber intakes showed suboptimal regular dietary intakes. While 61% were within range for percent calories from fat, 28% met recommended fiber intakes, and only one participant met fruit and vegetable recommendations [38,48]. There were no differences in FFQ intakes by gender or BMI category. The majority of participants consumed pasta (88%) and pulses (56%) on a monthly basis. Sixty-three percent stated they did not purposefully eat gluten-free foods. Supplemental Table S1 shows the mean nutrient intakes for the 24 h food logs by treatment meal type for the pre-test days. There were no mean differences between treatments in participant nutrient intake.

### 3.4. Subjective Appetite Measures

**Figure 2:**
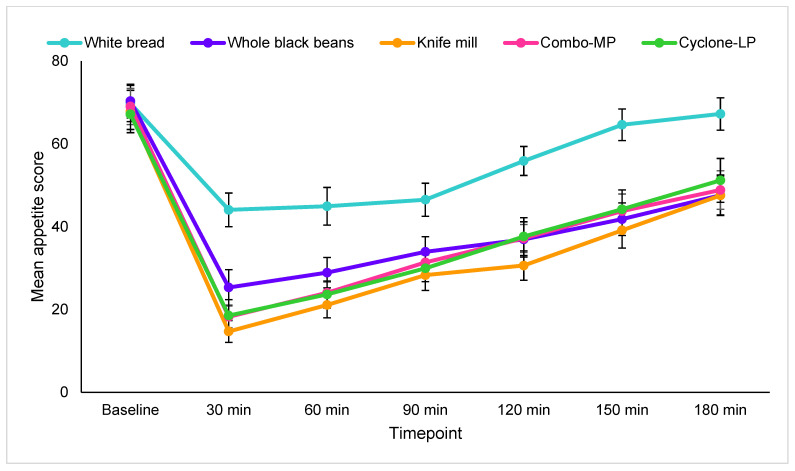
Mean appetite scores among study participants (*n* = 18).

Main time by treatment effects were significant for hunger [F(11.0, 214.0) = 2.0, *p* = 0.03], prospective consumption [F(13.0, 272.6) = 2.0, *p* = 0.02], and average appetite score [F(10.2, 212.1) = 2.3, *p* = 0.01]. Trending effects were evident for satiation [F(14.0, 293.1) = 1.7, *p* = 0.053] and desire to eat [F(13.7, 288.1) = 1.6, *p* = 0.085]. Post hoc analyses indicated lower overall appetite, hunger, and prospective consumption after whole beans and the three bean pasta treatments than after white bread. Satiety, conversely, was significantly greater with these four dietary treatments than with white bread. No appetite measure differences were observed between whole black beans and any of the pasta treatments. Individual appetite scores for each treatment by time increments are shown in Supplemental Table S2. Average appetite summary scores indicated that the whole black bean control (*p* = 0.01) and the three black bean pastas (Knife mill *p* = 0.003, Combo-MP *p* = 0.003, Cyclone-LP *p* = 0.003) were more appetite suppressing than the white bread control (Figure 2).

### 3.5. Sensory

Most sensory measures, including aroma, flavor, and meal size did not vary between control treatments or the three bean pastas (Table 3). Post hoc data indicated that appearance of the whole black bean meal was significantly less favorable in comparison to the other four treatments. Participants also ranked the whole black bean meal texture significantly lower than that of the white bread, Knife mill pasta, and Cyclone-LP pasta, but not the Combo-MP pasta. The mean score for the pasta sauce alone is shown in Table 3, but it was not compared statistically since the meals all contained it. The mean score for likelihood of purchasing the three pasta types was 5.76 (neutral–slightly likely), with no differences between them (data not shown).

### 3.6. Gastrointestinal Symptoms

There were no differences in participant-reported bloating, flatulence frequency, stool frequency, or stool consistency (Table 4).

### 3.7. Postprandial Glucose Response

**Figure 3:**
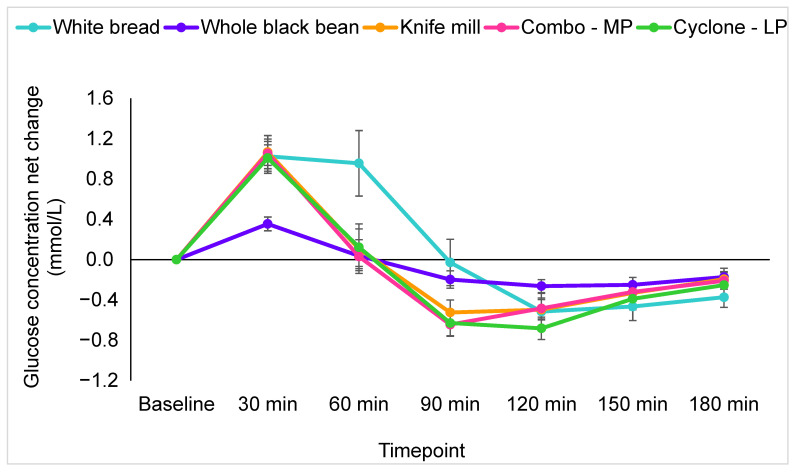
Incremental changes in plasma glucose for all treatments. Values are means plus standard error of the mean for change from baseline (mmol/L).

Results for glucose are presented as net change from fasting values. There was no significant difference between fasting blood glucose or insulin concentrations on different test days by individual. Plasma glucose concentrations fluctuated by study treatment over the 180 min testing period [F(11.6, 238.2) = 4.1, *p* < 0.001] (Figure 3). However, no significant differences were observed in post hoc analyses (*p* > 0.05).

**Figure 4:**
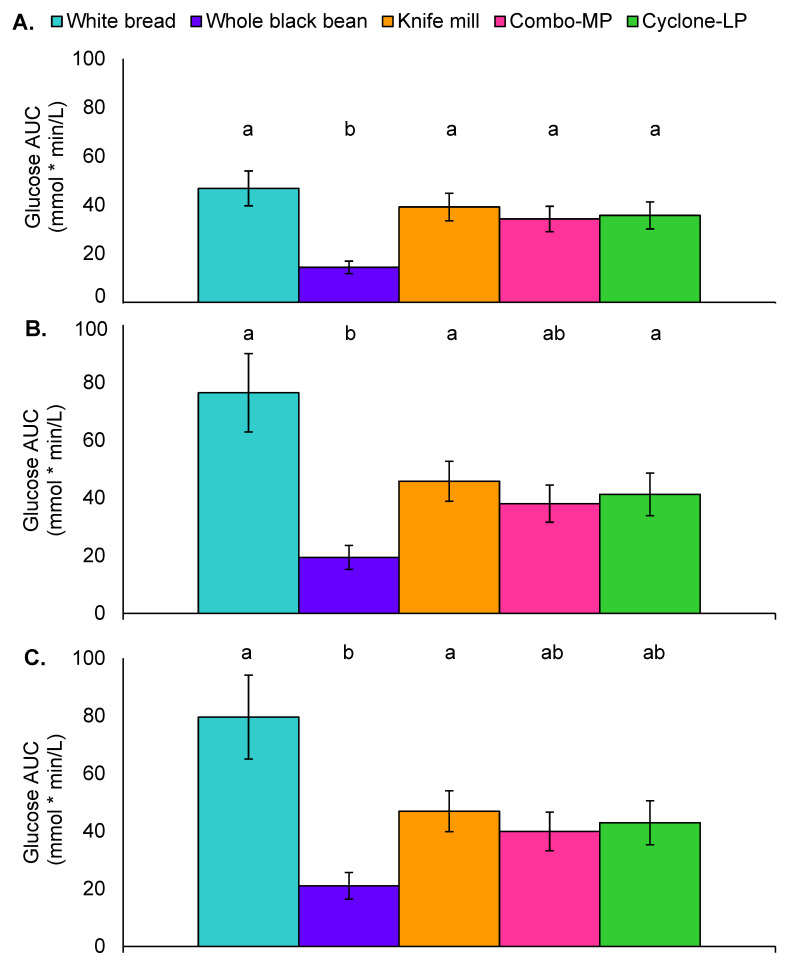
Glucose area under the curve by treatment: (**A**) 60 min, (**B**) 120 min, (**C**) 180 min. Values represent incremental area under the curve (iAUC) and standard error of the mean (mmol * min/L). Letters that differ indicate significant differences (*p* < 0.05) between treatments.

Glucose response was evaluated via iAUC from baseline to 60 min, baseline to 120 min, and baseline to 180 min (Figure 4). Univariate ANOVA indicated significant differences in glucose response at all durations ([F(4, 80) = 5.8, *p* < 0.001], [F(4, 80) = 5.0, *p* = 0.001], [F(4, 81) = 4.2, *p* = 0.004]). Post hoc analyses showed that, from 0–60 min, glucose iAUC was greater with the white bread treatment as compared to whole black beans (*p* < 0.001). Whole black bean glucose iAUC was also significantly lower than the Knife mill (*p* = 0.004), Combo-MP (*p* = 0.01), and Cyclone-LP pastas (*p* = 0.002) (Figure 4). Similar findings were observed for the 0–120 min and 0–180-min durations. White bread glucose iAUC was greater than whole black beans (*p* < 0.001) and whole black beans had a lower 0–120 min glycemic response than Knife mill pasta (*p* = 0.02) and Cyclone-LP pasta (*p* = 0.02), and trended lower than Combo-MP pasta (*p* = 0.07) (Figure 4B). From 0–180 min, white bread (*p* = 0.001) and Knife mill pasta (*p* = 0.047) produced a significantly greater glucose iAUC than whole black beans. The whole black bean control also trended lower than Cyclone-LP pasta (*p* = 0.08) (Figure 4C).

### 3.8. Postprandial Insulin Response

**Figure 5:**
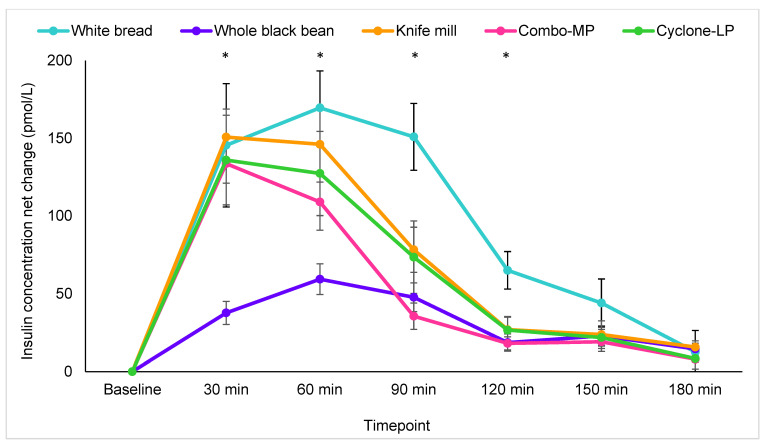
Incremental changes in serum insulin for all treatments with mean imputation. Values are means plus standard error of the mean for change from baseline (pmol/L). * *p* < 0.05.

Time by treatment effects were also present for serum insulin (Greenhouse–Geisser correction F(16.8, 214.4) = 4.3, *p* < 0.001) (Figure 5). Post hoc analyses indicated that these differences were evident between white bread and whole black beans (*p* = 0.003) and Combo-MP pasta (*p* = 0.01). Although not significant, serum insulin response trended lower with Cyclone-LP pasta than with the white bread control (*p* = 0.06).

**Figure 6:**
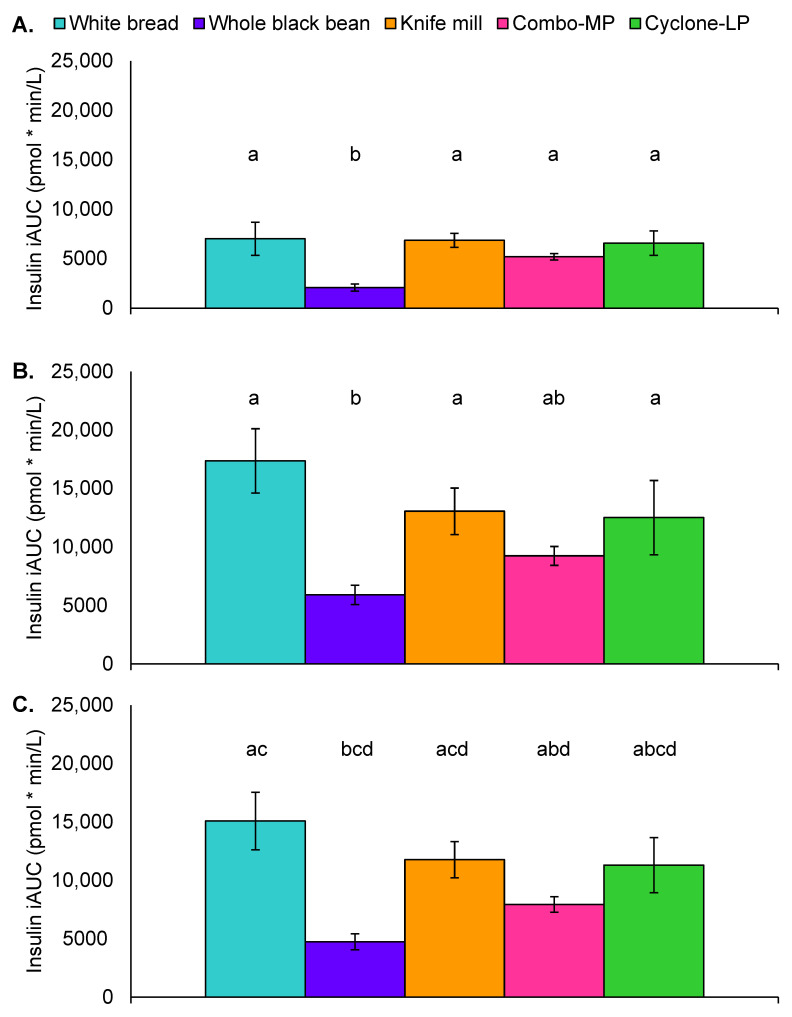
Insulin area under the curve by treatment: (**A**) 60 min, (**B**) 120 min, (**C**) 180 min. Values represent incremental area under the curve (iAUC) and standard error of the mean (pmol * min/L). Letters that differ indicate *p* < 0.05 between treatments.

Univariate ANOVA indicated significant main effect differences at 0–60 min [F(4, 60) = 9.6, *p* < 0.001], 0–120 min [F(4, 61) = 6.2, *p* < 0.001], and 0–180 min [F(4, 51) = 6.1, *p* < 0.001]. Between 0 and 60 min, insulin iAUC was greater with white bread than with whole black beans (*p* < 0.001). Whole black bean insulin iAUC was significantly lower than the three black bean pastas (all *p* < 0.001) (Figure 6A). Insulin iAUC was also higher for white bread than whole black beans between 0–120 min and between 0–180 min (both *p* < 0.001). Insulin iAUC from 0–120 min was also significantly lower for whole black beans than Knife mill pasta (*p* = 0.003), Cyclone-LP pasta (*p* = 0.001), and trended lower than Combo-MP pasta (*p* = 0.07). The Combo-MP pasta also produced a lower insulinemic response than white bread, and whole black beans were lower than Knife mill pasta (Figure 6A–C).

## 4. Discussion

The metabolic differences observed between whole beans and black bean pastas may be due to the intact cell wall structure of the whole beans, or the food matrix. Plant foods that are left in their original form blunt one’s glycemic and insulinemic response [50] as compared to those that have undergone food processing such as chopping, grinding, and flour formation [51,52]. Food processing can increase surface area and subsequent ability for human digestive enzymes to access energy from foods that have been extracted from their original source (e.g., whole almonds versus almond butter). However, additional study is needed to evaluate differences in metabolic response to modified pulses and legumes intended for use in food products. Currently, there is no standardization process used in pulse flour creation [35].

When addressing glycemic response, the three black bean pastas behaved similarly. The Knife mill had slightly larger particles than Cyclone-LP and Combo-MP. The Cyclone-LP pasta had 2 g less fiber and 5 g less protein than the Knife mill and Combo-MP pastas. Since no differences were observed, this suggests variation in particle size, and fiber and protein were not biologically meaningful in normoglycemic adults. All three bean pastas were formed into spaghetti with comparable compact structures which could account for the similarities in glycemic response [53].

Subjective appetite measures were lower with black bean pasta consumption as compared to the white bread control. However, there were no differences between the three pastas in any appetite metric. Previous studies conducted among young adults have also reported an absence of satiety differences with pastas of variable nutrient content. In a randomized double-blind crossover trial, participants did not report greater satiety with high protein or high fiber pasta than with refined wheat control pasta [54]. Participants could not differentiate between whole grain and refined grain pastas when appetite or hunger ratings were assessed in another randomized control crossover study [55].

Study participants did not report significant changes in gastrointestinal symptoms by treatment type. Although the FFQ results indicated that 73% of the participants consumed less than 20 g of fiber per day, the average fiber intake from the 24 h recalls was 20.5 g. This amount is similar to the fiber content of the whole black beans and black bean pastas. It is possible that gastrointestinal issues were minimal among this sample due to fiber intakes that were higher than the US national average. The current study findings and our previous work suggest that consumption of 100% black bean pastas would not increase GI symptoms in other healthy adults [42].

Participants indicated that the bean pastas were acceptable to them in a sensory perception capacity. In a 2020 survey of college students, almost 50% reported having eaten pulse pastas. Over 10% of these students consumed them at least once a month [56]. Pasta, in general, is a commonly consumed food in the US and one that can be health promoting. In another study, pasta consumers ate more fiber, food folate, and certain minerals than non-consumers [3]. However, the type of pasta consumed matters. In a separate cross-sectional study, diet quality was better among those who ate pasta noodles compared to non-consumers, while the reverse was true for individuals who primarily consumed pasta in the form of macaroni and cheese [57]. Bean pastas are even more nutrient dense than wheat pastas and offer a convenient method to increase pulse consumption, something that is lacking in many US households. Increased consumption of GF foods also makes bean pastas a desirable food product.

This study is the first to evaluate 100% black bean pasta intake on metabolic response, subjective appetite measures, and gastrointestinal symptoms among healthy adults. Additionally, this study was a randomized crossover with two control treatments—a positive and a negative—a combination that is rarely used in dietary intervention studies conducted among human subjects. However, while this study has many strengths, limitations to our study design and research findings are acknowledged. The study sample included young adults without obvious metabolic aberrations. Therefore, we cannot extrapolate our findings to individuals with T2DM or older free-living adults. We also tested study treatments in an acute setting. Further research is needed to evaluate the metabolic response to 100% bean pastas using a longitudinal study design.

## 5. Conclusions

This study confirms that, as expected, milling-related structural alterations in 100% black bean pastas produce a significant change in blood glycemic response in comparison to cooked whole black beans in healthy young adults. However, the milling techniques that produced varying amounts of protein in the pasta flours did not alter their glycemic and insulinemic responses. This study demonstrates the need to study biomarkers related to bean and other pulse ingredients to develop products with desired health outcomes. Black bean pastas are palatable, easy to consume functional foods that can help increase pulse consumption in the population.

## Figures and Tables

**Table 1:** Particle size distribution of whole black bean flours ^1^.

Mill Type	d (0.1) µm	d (0.5) µm	d (0.9) µm	MV µm
Knife mill	11.3 ^a^	62.4 ^a^	251.3 ^a^	104.2 ^a^
Compression/Decompression				
Combo-MP	2.4 ^c^	16.7 ^c^	33.4 ^b^	17.2 ^c^
Cyclone-LP	2.8 ^b^	20.4 ^b^	52.3 ^b^	26.2 ^b^

^1^ Data shown for d (0.1), d (0.5), and d (0.9) correspond to the maximum diameters of 10%, 50%, and 90% of the particles, respectively (in % of total volume). Results are means of triplicate analysis for each bean flour type. Values followed by different superscript letter in the same column are significantly different (*p* < 0.05). MV represents the mean diameter of the volume distribution.

**Table 2:** Nutrient composition of test meals.

Characteristics	White Bread	Whole Black Beans	Knife Mill	Combo-MP	Cyclone-LP
Total weight (g)	111.5	409.6	451.5	450.5	417.4
Pasta Sauce (g)	-	213.0	213.0	213.0	213.0
Bread (g)	111.5	-	-	-	-
Pulses (g)	-	196.6	238.5	237.5	204.4
Energy (kcal)	289.4	445.8	426.0	440.3	388.2
Total Carbohydrate (g)	52.6	78.2	72.1	72.4	69.5
Fiber (g)	2.6 ^a^	28.2 ^b^	22.1 ^b^	22.4 ^b^	19.5 ^b^
Available CHO (g)	50.0	50.0	50.0	50.0	50.0
Pasta Sauce (g)	-	20.0	20.0	20.0	20.0
Bread (g)	50.0	-	-	-	-
Pulses (g)	-	30.0	30.0	30.0	30.0
Protein (g)	10.2 ^a,c^	20.9 ^b,d^	19.5 ^b,d^	20.5 ^b,d^	13.7 ^b,c^
Fat (g)	4.3	5.4	6.5	7.5	6.1
Total Starch (g)	32.6	21.5	22.0	21.1	20.8

CHO = carbohydrate, g = grams, kcal = kilocalorie. Superscript letters that differ were significantly different from one another *p* < 0.05.

**Table 3:** Mean sensory evaluation scores for meals and pasta sauce (n = 18) ^1^.

Meal	Appearance	Aroma	Flavor	Texture	Meal Size
White bread control	5.5 ± 1.5 ^a^	6.1 ± 1.1	6.4 ± 1.3	6.6 ± 1.3 ^a^	4.8 ± 1.6
Whole black beans ^2^	3.9 ± 1.2 ^b^	5.6 ± 1.0	5.6 ± 1.2	4.6 ± 1.4 ^b^	5.5 ± 1.4
Knife mill	5.3 ± 2.0 ^a^	6.1 ± 1.0	6.3 ± 2.2	5.9 ± 1.9 ^a,c^	5.9 ± 1.3
Combo-MP ^3^	5.4 ± 2.0 ^a^	6.3 ± 1.5	6.1 ± 1.8	5.5 ± 2.0 ^a,b^	5.7 ± 1.0
Cyclone-LP	5.4 ± 1.7 ^a^	6.5 ± 1.2	6.3 ± 1.6	5.8 ± 1.6 ^a,c^	5.5 ± 1.0
Pasta sauce alone	---	---	6.6 ± 1.7	6.6 ± 1.4	---

^1^ All values are means ± standard deviation of the mean (SD). LP = low protein, MP = medium protein. Subscript letters that differ indicate significant differences (*p* < 0.05) between treatments; ^2^ *n* = 16, ^3^ *n* = 17.

**Table 4:** Gastrointestinal response symptoms among study participants (%, *n*) ^1^.

	Pre-Test	White Bread	Black Beans	Knife Mill	Combo-MP	Cyclone-LP
***Flatulence***						
No Change	83.8 (62)	86.7 (13)	46.7 (7)	46.7 (7)	44.4 (8)	70.6 (12)
Increased	12.2 (9)	13.3 (2)	46.7 (7)	46.7 (7)	50.0 (9)	29.4 (5)
Decreased	4.1 (3)	0	6.7 (1)	6.7 (1)	5.6 (1)	0
***Bloating***						
No Change	85.1 (63)	73.3 (11)	66.7 (10)	71.4 (10)	83.3 (15)	76.5 (13)
Increased	13.5 (10)	26.7 (4)	33.3 (5)	14.3 (2)	16.7 (3)	23.5 (4)
Decreased	1.4 (1)	0	0	14.3 (2)	0	0
***Stool frequency***						
No Change	82.8 (53)	78.6 (11)	73.3 (11)	53.3 (8)	77.8 (14)	88.2 (15)
Increased	7.8 (5)	7.1 (1)	13.3 (2)	40.0 (6)	16.7 (3)	11.8 (2)
Decreased	9.4 (6)	14.3 (2)	13.3 (2)	6.7 (1)	5.6 (1)	0
***Stool consistency***						
No Change	89.1 (57)	85.7 (12)	93.3 (14)	73.3 (11)	77.8 (14)	88.2 (15)
More Loose	9.4 (6)	0	6.7 (1)	26.7 (4)	11.1 (2)	5.9 (1)
More Firm	1.6 (1)	14.3 (2)	0	0	11.1 (2)	5.9 (1)

^1^ Data are the percentage and number of cases indicating yes for a given question. LP = low protein, MP = medium protein.

## Data Availability

The data presented in this study are available on request from the corresponding author. The data are not publicly available at this time due to ongoing analysis for a secondary research topic.

## Supplementary Materials

The following are available online at https://www.mdpi.com/article/10.3390/foods11111652/s1, Table S1: Macronutrient composition of dietary intake 24 h prior to test days; Table S2: Subjective appetite individual question scores and overall appetite score for all treatments.
