# Supplementary material for: Black Bean Pasta Meals with Varying Protein Concentrations Reduce Postprandial Glycemia and Insulinemia Similarly Compared to White Bread Control in Adults
Source: Foods. 2022 Jun 3;11(11):1652. doi: 10.3390/foods11111652 (PMC9180063; doi:10.3390/foods11111652)
Supplement: Supplementary file 1 [file foods-11-01652-s001.zip › foods-1695839-SI.pdf]

Table S1. Macronutrient composition of dietary intake 24 hours prior to test days[1]

Nutrient	White bread	Whole black bean[a]	Knife mill[b]	Combo - MP	Cyclone – LP
Energy (kcal)	2084.1 ± 158.1<br>(1254.6 - 3520.6)	1874.5 ± 150.6<br>(1042.8 - 3052.1)	2070.0 ± 151.9<br>(1472.0 - 3905.1)	1826.2 ± 135.4<br>(905.0 - 2796.2)	2012.2 ± 129.1<br>(1107.3 - 2820.0)
Carbohydrate (g)	241.5 ± 15.5<br>(166.1 - 420.2)	222.3 ± 18.8<br>(113.2 - 378.5)	248.8 ± 18.7<br>(152.2 - 414.4)	215.3 ± 14.4<br>(96.1 - 320.5)	243.3 ± 17.2<br>(99.9 - 363.5)
Available CHO (g)	221.1 ± 15.4<br>(138.2 - 399.1)	202.5 ± 18.2<br>(100.4 - 353.1)	227.4 ± 17.9<br>(133.4 - 386.8)	196.7 ± 13.8<br>(85.3 - 298.5)	220.7 ± 16.0<br>(87.0 - 340.6)
Total fiber (g)	20.5 ± 1.7<br>(10.0 - 35.1)	19.8 ± 1.7<br>(11.0 - 36.1)	21.4 ± 1.9<br>(10.5 - 39.8)	18.6 ± 1.6<br>(10.8 - 41.0)	22.6 ± 2.6<br>(10.0 - 57.3)
Total fat (g)	87.5 ± 9.9<br>(39.3 - 204.8)	77.6 ± 8.3<br>(39.0 - 136.4)	78.7 ± 7.5<br>(44.6 - 153.6)	72.8 ± 7.3<br>(30.4 - 123.3)	76.5 ± 7.1<br>(29.2 - 135.0)
Saturated fat (g)	28.6 ± 2.6<br>(11.6 - 55.4)	24.9 ± 2.9<br>(9.8 - 45.8)	26.1 ± 2.8<br>(13.0 - 56.3)	24.5 ± 2.8<br>(7.9 - 41.7)	25.0 ± 2.5<br>(10.6 - 41.6)
Cholesterol (mg)	240.2 ± 41.0<br>(46.3-639.8)	222.9 ± 44.9<br>(70.0 - 639.8)	223.2 ± 34.6<br>(99.5 - 619.5)	296.2 ± 53.4<br>(65.0 - 878.9)	253.1 ± 39.8<br>(65.0 - 639.8)
Sodium (mg)	3907.0 ± 334.0<br>(1578.3-7800.0)	3877.8 ± 376.4<br>(2239.5 - 7305.8)	3630.4 ± 312.9<br>(2078.7 - 6866.7)	3712.7 ± 274.1<br>(1492.2 - 5440.1)	4960.4 ± 935.6<br>(2244.5 - 19530.3)
Potassium (mg)	1619.9 ± 130.9<br>(860.0-2846.3)	1630.0 ± 157.5<br>(860.0 - 3102.1)	1792.2 ± 137.6<br>(861.7 - 2858.7)	1585.2 ± 107.9<br>(861.7 - 2580.3)	1768.5 ± 133.6<br>(898.2 - 3048.5)
Protein (g)	87.3 ± 6.0<br>(49.0-137.8)	78.1 ± 6.8<br>(37.9 - 119.2)	90.6 ± 6.1<br>(54.2 - 157.9)	82.3 ± 5.7<br>(37.3-117.8)	89.1 ± 5.8<br>(50.3 - 134.1)

[1] All values are means ± standard error of the mean (SEM) (range) CHO = carbohydrate, kcal = kilocalorie, g = gram, mg = milligram

[a]n = 16, [b]n = 17

Table S2. Subjective appetite individual question scores and overall appetite score for all treatments.[1]

	Baseline	30 minutes	60 minutes	90 minutes	120 minutes	150 minutes	180 minutes
Hunger							
White bread	65.1 ± 6.1	39.0 ± 5.0	41.2 ± 5.1	42.4 ± 4.7	50.4 ± 4.4	61.7 ± 5.2	63.9 ± 5.5
Whole black beans	68.2 ± 4.8	22.2 ± 4.5	25.1 ± 3.3	28.9 ± 3.8	31.1 ± 4.3	34.6 ± 4.4	42.8 ± 5.3
Knife mill	62.5 ± 6.5	12.2 ± 3.2	17.0 ± 2.9	24.1 ± 3.8	28.7 ± 4.5	33.0 ± 4.5	44.3 ± 5.4
Combo mill - MP	65.2 ± 5.3	12.3 ± 1.9	18.8 ± 2.1	27.9 ± 3.9	33.4 ± 4.0	40.3 ± 4.8	47.4 ± 5.1
Cyclone mill - LP	62.0 ± 5.2	14.5 ± 4.2	20.0 ± 3.4	26.5 ± 3.4	33.9 ± 5.0	40.0 ± 5.2	47.9 ± 6.2
Fullness							
White bread	18.7 ± 4.2	51.4 ± 5.3	51.4 ± 4.9	48.9 ± 4.6	39.3 ± 4.4	35.9 ± 5.8	27.4 ± 4.3
Whole black beans	23.1 ± 4.9	71.1 ± 4.8	67.1 ± 4.8	63.6 ± 5.1	56.6 ± 5.3	51.4 ± 5.2	46.7 ± 6.0
Knife mill	18.9 ± 5.0	80.9 ± 3.0	75.6 ± 3.5	68.6 ± 4.2	67.2 ± 4.2	60.0 ± 5.0	51.8 ± 5.4
Combo mill - MP	21.1 ± 4.5	76.1 ± 3.9	72.7 ± 3.4	64.3 ± 3.9	58.3 ± 4.5	52.3 ± 4.5	46.1 ± 5.3
Cyclone mill - LP	22.1 ± 4.8	77.9 ± 3.8	69.9 ± 5.3	67.7 ± 4.1	58.1 ± 5.1	51.6 ± 5.5	45.3 ± 5.9
Satiation							
White bread	26.3 ± 5.6	52.1 ± 3.9	49.2 ± 4.9	46.2 ± 4.6	40.7 ± 4.2	39.3 ± 5.8	33.1 ± 4.8
Whole black beans	21.2 ± 4.9	69.6 ± 4.1	65.9 ± 4.4	65.3 ± 3.9	58.5 ± 4.9	57.0 ± 3.8	54.3 ± 4.8
Knife mill	24.0 ± 5.8	80.3 ± 2.5	72.7 ± 5.0	70.5 ± 3.2	67.4 ± 3.9	61.4 ± 3.7	54.2 ± 4.4

Combo mill - MP	24.0 ± 5.1	74.4 ± 4.2	70.2 ± 3.5	65.6 ± 3.1	62.3 ± 3.6	52.4 ± 4.5	52.9 ± 4.6
Cyclone mill - LP	28.7 ± 4.7	78.6 ± 3.0	70.5 ± 3.6	64.9 ± 4.1	63.3 ± 4.4	55.3 ± 5.6	49.6 ± 6.1
Desire to eat							
White bread	66.8 ± 6.4	44.5 ± 5.3	46.1 ± 5.9	46.7 ± 4.8	56.2 ± 4.8	67.6 ± 5.3	66.9 ± 4.7
Whole black beans	66.6 ± 4.9	24.8 ± 5.3	26.7 ± 4.2	35.8 ± 4.1	34.7 ± 5.0	40.2 ± 4.9	48.0 ± 5.4
Knife mill	62.6 ± 7.0	11.3 ± 2.6	20.9 ± 4.1	26.3 ± 4.6	27.1 ± 3.9	39.2 ± 5.0	47.9 ± 5.4
Combo mill - MP	66.0 ± 5.8	16.0 ± 3.5	20.7 ± 3.5	29.6 ± 3.6	35.4 ± 4.1	41.8 ± 5.0	47.9 ± 5.1
Cyclone mill - LP	61.6 ± 4.7	17.2 ± 4.7	20.0 ± 4.2	27.5 ± 4.2	36.0 ± 5.4	44.3 ± 5.4	51.8 ± 5.9
Prospective consumption							
White bread	66.0 ± 3.8	44.3 ± 3.5	43.9 ± 4.0	45.9 ± 4.2	56.3 ± 3.1	65.1 ± 3.8	65.6 ± 3.6
Whole black beans	69.7 ± 3.3	25.5 ± 4.3	30.9 ± 3.8	34.6 ± 3.7	38.6 ± 4.3	43.9 ± 3.9	46.3 ± 4.6
Knife mill	65.1 ± 4.7	16.3 ± 3.0	22.1 ± 3.5	31.4 ± 4.2	33.9 ± 4.1	44.4 ± 4.8	49.9 ± 4.6
Combo mill - MP	66.0 ± 3.9	20.8 ± 3.1	29.4 ± 3.6	32.3 ± 3.4	38.1 ± 3.3	45.3 ± 4.0	46.3 ± 4.6
Cyclone mill - LP	67.1 ± 3.5	20.4 ± 3.7	24.5 ± 3.3	33.6 ± 3.7	38.7 ± 4.0	44.1 ± 4.3	50.4 ± 4.5
Average appetite score							
White bread	69.8 ± 4.4	44.1 ± 4.1	44.9 ± 4.5	46.5 ± 4.0	55.9 ± 3.5	64.6 ± 3.8	67.2 ± 3.9
Whole black beans	70.3 ± 4.1	25.3 ± 4.3	28.9 ± 3.7	33.9 ± 3.7	36.9 ± 4.3	41.8 ± 3.9	47.6 ± 4.9
Knife mill	67.8 ± 5.1	14.7 ± 2.6	21.1 ± 3.1	28.3 ± 3.7	30.6 ± 3.5	39.1 ± 4.3	47.6 ± 4.7
Combo mill - MP	69.0 ± 4.4	18.3 ± 2.6	24.0 ± 2.6	31.4 ± 3.0	37.2 ± 3.4	43.8 ± 4.1	48.9 ± 4.7
Cyclone mill - LP	67.1 ± 3.7	18.5 ± 3.9	23.6 ± 3.3	30.0 ± 3.2	37.6 ± 4.6	44.2 ± 4.7	51.2 ± 5.3
